# Incidence Density and Risk Factors of Diabetic Retinopathy Within Type 2 Diabetes: A Five-Year Cohort Study in China (Report 1)

**DOI:** 10.3390/ijerph120707899

**Published:** 2015-07-10

**Authors:** Lei Liu, Jingyang Wu, Song Yue, Jin Geng, Jie Lian, Weiping Teng, Desheng Huang, Lei Chen

**Affiliations:** 1 Department of Ophthalmology, The First Affiliated Hospital of China Medical University, Shenyang, 110001, China; E-Mails: liuleijiao@163.com (L.L.); happygirl860824@163.com (J.W.); yuesong567@163.com (S.Y.); asdaka_1@163.com (J.G.); 2 Department of Healthcenter, Fengyutan Community, Shenyang, 110014, China; E-Mail: lianjie08147@163.com; 3 Key Laboratory of Endocrine Diseases in Liaoning Province, The First Hospital of China Medical University, Shenyang, 110001, China; E-Mail: twp@vip.163.com; 4 Department of Epidemiology, School of Public Health, China Medical University, Shenyang, 110122, China

**Keywords:** community, diabetic retinopathy, epidemiology, incidence density, risk factors

## Abstract

A prospective study was carried out from August 2010 to August 2014 in the community of Fengyutan (China). Six hundred and twenty two T2D subjects were collected. The incidence density of diabetic retinopathy (DR) was 1.81% person-year (95% confidence interval, CI: 1.21–2.43% person-year). After a Cox regression model analysis and adjusted confounding factors, independent predictors related to the incidence of DR including male gender (adjusted hazard ratios, aHR: 1.47, 95% CI: 1.21–1.62), with hypertension (aHR: 1.49, 95%: 1.12–1.73), duration of diabetes > 10 years (aHR: 2.28, 95%: 2.05–2.42), uncontrolled diabetes (aHR: 1.76, 95%: 1.41–2.01), total cholesterol ≥ 200 mg/dL (aHR: 1.54, 95%: 1.34–1.72) and HbA1c ≥ 7% (mmol/mol) (aHR: 2.12, 95%: 1.87–2.32). Duration of T2D revealed the significantly dose-response relationship to the onset of DR. The incidence density of DR in the Chinese community was relatively low in comparison with other studies. More attention should be paid to the T2D patients, especially of male gender, with hypertension, longer duration of diabetes, uncontrolled diabetes, total cholesterol ≥ 200mg/dL and HbA1c ≥ 7% (mmol/mol).

## 1. Introduction

Diabetic retinopathy (DR) has been one of the foremost causes of blindness in the working age group [1]. DR is a priority disease in the “VISION 2020” initiative for the global elimination of avoidable blindness. The World Health Organization (WHO) has recommended its member countries to integrate a program approach for DR within their prevention of blindness programs. DR and age-related macular degeneration (AMD) play an important role in causes of visual impairment in elderly Chinese [2]. With the increasing number of diabetes and pre-diabetes cases in China, the prevalence of DR would be also increasing [3,4]. To date, epidemiological research on DR in China has been limited to prevalence estimates from cross-sectional studies only [5,6]. The number of studies on community based prospective epidemiology in DR is limited. The aims of this study were to evaluate the risk factors of DR in patients with type 2 diabetes (T2D), and to establish the incidence density of new DR in these patients over time. Consequently, it would be useful to early detection and prevention of DR in China.

## 2. Materials and Methods

### 2.1. Study Population

This study was carried in the healthcare centre of Fengyutan community (5.5 km^2^ and 80,177 inhabitants in 2010) which was a demonstration unit for the prevention and treatment model for diabetic eye disease at the Liaoning Diabetic Eye Center. The study has recently been described in detail [7]. In 2010, there were 945 T2D health files in Fengyutan. Type 2 diabetes were diagnosed by primary care physicians at the Fengyutan healthcare centre according WHO criteria and then the health files were recorded. The research was carried out from August 2010 to August 2014 based on screening for DR of elderly (over 60 years of age) residents in Fengyutan community. The inclusion criteria were set as: confirmed Type 2 diabetes, with health files at the Fengyutan healthcare center, who were willing to participate in this cohort study and who provided informed consent for this study. Excluding ineligible patients with T2D who had died, moved out of the community, were hospitalised or institutionalised in nursing homes, with concomitant ocular disease other than DR, history of ocular trauma and intraocular surgery, a total of 735 patients with health files were recruited. Briefly, a total of 548 T2D patients without DR (mean age 69.09 ± 3.18 years, range 60–82) (response rate 74.56%) participated in this cohort study in 2010. During these years, patients were assessed annually.

### 2.2. Data Collection

The general information for T2D patients including name, age, gender, and history for disease including hypertension, family history of diabetes, diabetic duration, and treatment for diabetes were acquired by questionnaire. Body examination data including blood pressure, height and weight were collected. Body mass index (BMI) was calculated by dividing weight in kilograms by height in meters squared (kg/m^2^). A venous blood sample was drawn from an antecubital vein in the morning after more 8 h of fasting for determination of total cholesterol, triglyceride, FPG levels and concentrations of HbA1c. All measurements were performed at the Endocrinology Laboratory, China Medical University, Shenyang, China, using commercially available assays.

### 2.3. Eye Examination

A comprehensive examination of each subject's eyelids, palpebral and bulbar conjunctiva, sclera and cornea, anterior chamber, iris and lens was carried out using a slit-lamp. The ocular pressure was measured by an applanation tonometer (Topcon, Tokyo, Japan). The pupils of patients were dilated by instilling one drop of 1.0% tropicamide with normal intraocular pressure. If the pupil could not dilate after 30 min, one more drop of 1.0% tropicamide was added to the previous one. Fundus examination was carried out using slit-lamp bio-microscope and + 90 D Volk lens at × 16 magnification by two ophthalmologists. Every subject was taken fundus photographs for diagnosis and following up.

### 2.4. Definition for Diagnosis

The diagnosis of diabetes was made with the criteria by World Health Organization. All of the subjects were T2D patients. The patient whose duration of diabetic was less than 1 year was considered as newly diagnosed diabetes. Duration of diabetes was the time interval between the date of diagnosis of diabetes and the date of eye examination. DR was graded using the modified Airlie House classification and the Early Treatment Diabetic Retinopathy Study retinopathy severity scheme [8,9].

### 2.5. Statistical Analysis

The incidence density of DR was evaluated by life table analysis. 95% confidence intervals (CI) for incidence density were calculated using the Poisson distribution. Mean ± SD was used for measurement data. In univariate analysis, a t-test was applied for continuous variables and chi-square test (X^2^) for nominal-scale data. The influence of T2D on the risk of DR was analyzed using a Multivariable Cox Regression. P-values less than 0.05 were considered statistically significant. All analyses were performed with Statistical Package for the Social Sciences software (Version 16.0, SPSS Inc., Chicago, IL, USA).

### 2.6. Ethics Statement

The mayor and the welfare section of Fengyutan community approved this study. The research followed the tenets of the Declaration of Helsinki and informed consents were obtained from the subjects after explanation of the nature and possible consequences of the study. The research was approved by Institutional Ethics Committee of the First Affiliated Hospital of China Medical University.

## 3. Results

A total of 622 subjects over 60 years of age (60–84 years old, average age 69 ± 5.87) with T2D were collected in 5 years. There were 304 (48.98%) males and 318 (51.02%) females. The results for subjects attended in the study and followed up for each year were shown in Table 1.

**Table 1:** Number of survival person-year using life table analysis for DR.

Year	No. of subjects at the beginning of the year	No. of subjects attending into the study during the year	Incidence DR during the year	Subjects without follow-up during the year	Incidence (person-year)
2010	548	40	9	20	548
2011	559	55	10	30	559
2012	574	50	8	35	574
2013	581	35	11	10	581
2014	595	20	14	9	595

Abbreviation: DR, diabetic retinopathy.

From Table 1 we could calculate the incidence density of DR which was 1.81% person-year (95%CI, 1.21–2.43% person-year). Characteristics of participants in the cohort study are shown in Table 2.

**Table 2:** Characteristics of participants in the cohort study, 2010 to 2014.

Characteristics	Diabetes (n = 622)	DR (n = 52)	*p*
Age (years)	69 ± 5.87	72 ± 4.11	< 0.01
Sex, male	48.98	51.92	< 0.01
Duration of diabetes (years)	5.04 ± 1.11	7.11 ± 2.01	< 0.01
With hypertension history	40.11	60.41	< 0.01
With family history of diabetes	20.12	42.35	< 0.01
Control diabetes	21.25	13.55	< 0.01
Systolic BP, mmHg	122.9 ± 17.6	139.1 ± 17.4	< 0.01
Diastolic BP, mmHg	78.1 ± 10.5	87.1 ± 10.4	< 0.01
Total cholesterol, mg/dL	188.3 ± 41.1	194.2 ± 35.7	< 0.01
Triglyceride, mg/dL	177.4 ± 101.3	147.5 ± 140.7	< 0.01
BMI (kg/m^2^)	23.8 ± 3.2	24.9 ± 3.1	< 0.01
HbA_1_c (% (mmol/mol))	5.8 ± 0.4	7.4 ± 1.4	< 0.01
FPG, mg/dL	92.5 ± 9.6	151.2 ± 45.5	< 0.01

Data are means ± SDs or frequency (%). Abbreviation: FPG: Fasting plasma glucose; BMI: Body mass index; HbA_1_c: Glycated hemoglobina1c; DR, diabetic retinopathy.

**Table 3:** Hazard ratios of the factors associated with onset of DR.

Variable	HR	HR 95%CI	*p*
Age (years)			
< 70	1		
≥ 70	1.36	0.97–1.56	< 0.01
Gender			
Female	1		
Male	1.79	1.31–2.32	< 0.01
Hypertension			
Without	1		
With	1.39	1.01–1.62	< 0.01
Duration of diabetes (years)			
< 5	1		
5–10	1.76	1.34–2.20	< 0.01
> 10	1.98	1.45–2.31	< 0.01
Family history of diabetes			
Without	1		
With	1.19	0.78–1.31	0.06
Controlled diabetes			
Yes	1		
No	1.67	1.32–2.13	< 0.01
Systolic BP, mmHg			
< 140	1		
≥ 140	1.14	0.78–1.42	0.08
Diastolic BP, mmHg			
< 90	1		
≥ 90	1.04	0.88–1.21	0.22
BMI (kg/m^2^)			
< 25	1		
25–27.4	1.08	0.88–1.32	0.26
> 27.4	1.19	1.08–1.24	0.03
FPG, mg/dL			
< 110	1		
110–200	1.12	0.85–1.32	0.11
> 200	1.92	1.75–2.31	< 0.01
Total cholesterol, mg/dL			
< 200	1		
≥ 200	1.38	1.17–1.68	< 0.01
Triglyceride, mg/dL			
< 150	1		
≥ 150	1.11	0.87–1.28	0.16
HbA_1_c (% (mmol/mol))			
< 7	1		
≥ 7	1.77	1.57–2.36	< 0.01

Abbreviation: CI: Confidence interval. FPG: Fasting plasma glucose; BMI: Body mass index; HbA_1_c: Glycated hemoglobina1c; DR, diabetic retinopathy.

Table 3 showed us that the risk factors for incidence of DR in T2D patients by a Multivariable Cox Regression. The highest hazard ratios (HR) was duration of diabetes mellitus (DM) longer than 10 years (HR = 1.98; 95% CI = 1.45–2.31). In addition, being male (HR = 1.79; 95% CI = 1.31–2.32) with hypertension (HR = 1.39; 95% CI = 1.01–1.62), uncontrolled diabetes (HR = 1.67; 95% C I= 1.32–2.13), BMI more than 27.4 kg/m^2^ (HR = 1.67; 95% CI = 0.88–1.32), fasting plasma glucose (FPG) more than 200 mg/dL (HR = 1.92; 95% CI = 1.75–2.31), total cholesterol more than 200 mg/dL (HR = 1.38; 95% CI = 1.17–1.68) and glycated haemoglobin (HbA1c) more than 7% (mmol/mol) (HR = 1.77; 95% CI = 1.57–2.36) were significant appeared to be incidence of DR.

After controlling for age, family history of diabetes, systolic blood pressure (BP), diastolic BP and triglyceride, as Table 4 shows, male gender, with hypertension, longer duration of diabetes, uncontrolled diabetes, total cholesterol ≥ 200 mg/dL and HbA1c ≥ 7% (mmol/mol) was significantly associated with incidence of DR. Duration of T2D revealed the significantly dose-response relationship to the onset of DR (X2-test for trend = 8.89, *p* < 0.05) after controlling for other factors.

**Table 4:** Adjusted hazard ratios of the factors associated with onset of DR.

Variable	aHR	HR 95%CI	*p*
Male gender	1.47	1.21–1.62	< 0.01
With hypertension	1.49	1.12–1.73	< 0.01
Duration of diabetes (years)*			
5–10	1.86	1.53–2.01	< 0.01
> 10	2.28	2.05–2.42	< 0.01
Uncontrolled diabetes	1.76	1.41–2.01	< 0.01
Total cholesterol, mg/dL ≥ 200	1.54	1.34–1.72	< 0.01
HbA_1_c (%) ≥ 7	2.12	1.87–2.32	< 0.01

*Duration of diabetes revealed the significantly dose-response relationship to the onset of DR (X^2^-test for trend = 8.89, *p* < 0.05) after controlling for other factors.

## 4. Discussion

After five years, the incidence density of DR was found to be of 1.81 cases per 100 person-years, lower than 6.62% observed in a larger and two years cohort in Kinmen, Taiwan [10]. Different incidence density rates might include different sample, study interval, age of subjects and different ethnic group. Other population-based studies reported that the incidence density DR ranged from 1.56% per year to 17.03% per year [11,12,13,14,15]. Due to differences in study populations, methodologies, and definition of DR, the estimation of incidence density of DR may be discordant among various researches. The other reason for the lower incidence density was that most DR subjects were patients with NPDR in our study. Except incidence density, the DR incidence rate was 46.89% in Shanghai of China [16].

Many studies investigated that the duration of diabetes was a strong risk factor for development of DR [17,18,19] according with our research. The Wisconsin Epidemiologic Study of Diabetic Retinopathy (WESDR) had revealed the prevalence of any retinopathy was 8% at duration of 3 years diabetes, 25% at duration of 5 years diabetes, 60% at duration of 10 years diabetes, and 80% at duration of 15 years diabetes. The prevalence of PDR was 0% at duration of 3 years diabetes and increased to 25% at duration of 15 years diabetes [20]. So annual retinal examination and early detection of DR could considerably reduce the risk of visual loss in diabetic individuals.

This study has suggested that arterial hypertension history was a independent risk factor for DR, it was the same with Australia [21], which would be because that hypertension was a risk factor for DR [22] and many diabetic always had hypotension, the renin-angiotensin system was activated by chronic hyperglycemia, and then the vitreous fluid level of angiotensin II (AII) was elevated in patients with PDR and diabetic macular edema. AII increased vascular permeability and promoted neovascularization. Recent studies had suggested that hypertension was a risk factor for the development and progression of DR and that blood pressure reduction could delay the progression of retinopathy [23]. It is worth noting that systolic BP and diastolic BP levels were not associated with incidence of DR in our research. The reasons for these outcomes should be made an intensive study in future.

Nearly study has revealed that poor glycemic control was a risk factor for DR [24], it was a community-based survey like this research. Because of many uncontrolled diabetes could not be confirmed from medical records, whose glycemic was always high, the incidence of development for DR would be increased. Because of preventing DR could through controlling systemic factors as intensive glycemic and blood pressure [25], so if we want to control the development of DR, we must control the system factors of diabetes.

As a predictor criterion for T2D controlling, the level of HbA1c also has been considered as an independent risk factor for the incidence of DR in our observation. This result is similar to another study that have longer screening interval [13]. In addition, the association between concentration of total cholesterol and onset of DR in this research supported previous outcomes from other population-based studies [26,27]. These results revealed that the association between factors and onset of DR including not only glucose levels, but also lipid concentration. T2D patients who had abnormal level of HbA1c or total cholesterol concentration should have fundus examinations more frequently in order to early detect incidence of DR.

Male gender was observed to be associated with the presence of any DR [28] as this study, but not its severity. Similar observations were made by Pradeepa *et al*., in an urban Indian population and in the Los Angeles Latino Eye Study [29,30]. The reason for this may be gene or life style. To our knowledge, as a five-year dynamic cohort study, this was the first prospective study based on five years follow-up in Chinese community for DR with elder T2D subjects. The incidence density was the number of new cases of a given disease during a given period in specified population. It was also used for the rate at which new events occur in a defined population. It was different from prevalence, which referred to all cases, new and old, in the population at a given time. This study provided robust epidemiological information regarding the risk factors for DR. From a preventive medicine viewpoint, primary prevention of DR should focus on risk factors which were also assessed by Cox regression model in this study responsible for the incidence of any DR.

Because the health files were newly constructed in Shenyang community, and follow-up work was much easier in elder people, this study was a limited sample. The other short coming for this study included this study was a population based study in community health care, so there were no fundus fluorescein angiography (FFA), visual field and optical coherence tomography (OCT) for assistant diagnosis. The study was conducted only in one community of Shenyang, so there is a selection bias. There were many patients with progression of DR in this study, but we did not investigate the characteristics and risk factors in this paper and those would be reported in future.

## 5. Conclusions

This study showed us that there were many DR in Chinese community and many newly onset of DR should be screened each year, so some effective preventing methods were needed to do. Many preventing model should been constructed to prevent DR and health files would be completed, big sample study would be done in future.

## Abbreviations

aHR: adjusted hazard ratios
CI: Confidence interval
HbA1c: Glycated hemoglobina1c
DR: diabetic retinopathy
